# In vivo pre-activation of monocytes in patients with axial spondyloarthritis

**DOI:** 10.1186/s13075-015-0694-2

**Published:** 2015-07-16

**Authors:** Kristina Conrad, Peihua Wu, Joachim Sieper, Uta Syrbe

**Affiliations:** Charité-Universitätsmedizin Berlin, CBF, Medizinische Klinik für Gastroenterologie, Infektiologie und Rheumatologie, Hindenburgdamm 30, Berlin, 12203 Germany; Deutsches Rheumaforschungszentrum, Charitéplatz 1, Berlin, 10117 Germany

## Abstract

**Introduction:**

Innate immune responses, including monocyte functions, seem to play an important role in the pathogenesis of axial spondyloarthritis (axSpA). Therefore, we characterized the phenotype and functional state of monocytes of patients with axSpA.

**Methods:**

Fifty-seven patients with axSpA, 11 patients with rheumatoid arthritis (RA), and 29 healthy controls were included in the study. We determined the percentage of classic, intermediate, and non-classic monocytes according to CD14 and CD16 expression and the expression of Toll-like receptor (TLR) 1, 2, and 4 in whole blood by flow cytometry. The percentage of monocytes producing interleukin (IL)-1beta, IL-6, tumor necrosis factor alpha (TNFα), IL-12/23p40, and IL-1 receptor antagonist (IL-1ra) was detected by flow cytometry after stimulation of whole blood without and with different TLR and nucleotide-binding oligomerization domain ligands—i.e., lipopolysaccharide (LPS), fibroblast-stimulating lipopeptid-1, PAM_3_CSK_4_, and muramyl dipeptide (MDP)—for 5 h. IL-10 production was measured after 18 h of stimulation in supernatants by enzyme-linked immunosorbent assay.

**Results:**

In patients with axSpA but not patients with RA, we found higher frequencies of classic monocytes than in controls (median of 90.4 % versus 80.4 %, *P* < 0.05), higher frequencies of monocytes spontaneously producing IL-1beta and IL-1ra (*P* < 0.05), and a higher percentage of monocytes producing IL-1beta after MDP stimulation (*P* < 0.05). Elevated cytokine production was confined to axSpA patients under conventional therapy (non-steroidal anti-inflammatory drugs) and not found in patients under TNFα inhibitor treatment. The LPS-induced production of IL-6 and IL-10 was lower in axSpA patients compared with controls (*P* < 0.05). Monocytic TLR expression was unaffected in patients with axSpA.

**Conclusion:**

Enhanced spontaneous and MDP-induced cytokine secretion by monocytes suggests *in vivo* pre-activation of monocytes in axSpA patients under conventional therapy which is reverted under TNF inhibitor treatment.

## Introduction

Axial spondyloarthritis (AxSpA) is the prototypic form of spondyloarthritis, a group of related diseases comprising AxSpA, reactive arthritis, arthritis related to inflammatory bowel disease, psoriasis, and uveitis [1]. AxSpA, nowadays subdivided into non-radiographic axSpA and ankylosing spondylitis (AS), is characterized by inflammation within the axial skeleton, predominately of the sacroiliac joints [1].

The pathogenesis and triggers of inflammation in axSpA are still unclear. AxSpA is strongly associated with the major histocompatibility complex (MHC) class I molecule HLA-B27; however, the involvement of CD8 T-cell responses could not be proven so far. In fact, in the HLA-B27 transgenic rat model, CD8 T cells are dispensable for disease induction [2]. Apart from presenting specific antigens to CD8^+^ T cells, HLA-B27 has the capability to induce endoplasmic reticulum stress [3]. In innate immune cells, this can trigger cytokine responses or modify the responsiveness of these cells to other stimulants, suggesting that innate immune functions might play a role in the pathogenesis of axSpA.

Monocytes belong to the innate, first-line defence against infections. They recognize bacterial structures by pattern recognition receptors, including Toll-like receptor (TLR) and nucleotide-binding oligomerization domain (NOD)-like-receptors. Ligation of TLR or NOD receptors rapidly triggers production of proinflammatory cytokines such as interleukin-1 (IL-1), IL-6, and tumor necrosis factor alpha (TNFα) in monocytes [4, 5]. Proinflammatory responses are tightly controlled by concomitant induction of anti-inflammatory mediators, such as IL-1ra, which competes with IL-1 for binding to the IL-1 receptor 1 [6], and IL-10, which is produced by monocytes at later time points after stimulation and downregulates TNFα production [7].

There are some reports that showed enhanced transcription and protein expression of inflammation-associated genes in blood monocytes of patients with AS, suggesting aberrant activation or disturbed responsiveness of monocytes in patients with axSpA [8, 9].

To further decipher potential involvement of monocytes in the pathogenesis of axSpA, we studied here the phenotype and function of monocytes from patients with axSpA. We performed a phenotypic analysis of monocytes and determined their spontaneous and TLR and NOD ligand-induced cytokine production.

## Methods

### Study subjects

Peripheral venous whole blood was collected in heparinised Vacutainer tubes (BD, Heidelberg, Germany) from 29 healthy donors (15/29 male, mean age of 35.3 ± 8.3 years) and from 57 patients with axSpA (39/57 male, mean age of 38.0 ± 10.6 years) meeting the Assessment of Spondyloarthritis International Society criteria for axSpA, of whom 39 patients (68.4 %) fulfilled the modified New York criteria for diagnosis of AS. Eleven patients (2/11 male, mean age of 59.7 ± 18.8 years) with rheumatoid arthritis (RA) were included as inflammatory controls. The number of patients and controls used for individual parts of the study (phenotypic analysis and cytokine analysis) is stated in each figure legend. Fifty-three (92.9 %) of the 57 patients with axSpA were HLA-B27-positive. Thirty-four patients with axSpA received conventional treatment with continuous or on-demand non-steroidal anti-inflammatory drugs (NSAIDs), and 23 patients received TNFα inhibitors. None of the patients with axSpA received disease-modifying anti-rheumatic drugs (DMARDs). Ten out of eleven patients with RA received prednisolone, eight out of 11 received synthetic DMARD therapy (four methotrexate, two hydroxychloroquine, one sulfasalacine, and one methotrexate plus sulfasalacine), and two patients received treatment with biologics (one TNFα inhibitor and one anti-IL-6 receptor antibody). Disease activity was determined according to the Bath ankylosing spondylitis disease activity index (BASDAI) [10]. C-reactive protein (CRP) was determined at the routine clinical laboratory of the Charité.

The study was approved by the local ethical commission of the Charité. All patients and control subjects gave consent to the study.

### Phenotypic characterization of peripheral blood monocytes

To analyse the expression of phenotypic surface markers, 2 ml of heparinized whole blood was incubated with 8 ml of erythrocyte lysing buffer (Qiagen, Hilden, Germany) on ice to lyse erythrocytes. The remaining cells were stained with the respective antibodies against CD14 (clone M5E2 PerCPcy5.5; BD), CD16 (clone eBioCB16 APC; ebioscience, Frankfurt, Germany), HLA-DR (clone L243 fluorescein isothiocyanate (FITC); BD Heidelberg, Germany), CD64 (clone 10.1 PE; Dako, Glostrup, DK), CD80 (clone 2D10 PE; biolegend, San Diego, CA, USA), CD115 (clone 12-3A3-1B10 PE; ebioscience), and CD163 (clone GHI/61PE; BD). For analysis of TLR expression, 50 μl of heparinized whole blood was incubated at room temperature with the respective antibodies against TLR1, i.e., CD281 (clone GD2.F4 TLR-1), TLR2, i.e., CD282 (clone TL2.1 TLR-2), and TLR4, i.e., CD284 (clone HTA125 TLR-4; all PE; ebioscience) in the dark for 15 min. Afterwards, erythrocytes were lysed with fluorescence-activated cell sorting (FACS) lysing solution (BD) and leukocytes were analysed by flow cytometry by using a FACS Calibur from Becton Dickinson (San Jose, CA, USA) and FlowJo 7.6.4 Software.

### In vitro stimulation of whole blood with Toll-like receptor and nucleotide-binding oligomerization domain ligands and intracellular cytokine detection

Heparinized peripheral blood (1 ml) was stimulated for 5 h without or with the TLR 1/2 ligand PAM_3_CSK_4_ (5 μg/ml), the TLR 2/6 ligand fibroblast-stimulating lipopeptid-1 (FSL-1; 1 μg/ml), the TLR 4 ligand lipopolysaccharide (LPS) (*Escherichia coli* O111:B4; 100 ng/ml), and the NOD2 ligand muramyl dipeptide (MDP) (2.5 μg/ml). All stimulants were purchased from invivoGen (San Diego, CA, USA).

To prevent cytokine secretion, 10 μg/ml brefeldin A (BFA) (Sigma-Aldrich, Steinheim, Germany) was added for the last 3 h of stimulation. After stimulation, erythrocytes were lysed with FACS lysing solution and the remaining cells were fixed by 2 % paraformaldehyde (Roth, Karlsruhe, Germany). Intracellular cytokines and CD68 were stained after permeabilization in 0.5 % saponin/phosphate-buffered saline buffer (Sigma-Aldrich, Steinheim, Germany) by using the following antibodies: anti-CD68-PerCPcy5.5 (clone Y1/82A; biolegend), anti-IL-12/23p40 (clone eBioHP40; e-bioscience, Frankfurt, Germany), anti-TNFα-FITC (clone Mab11; BD), anti-IL-1β-Alexa Fluor 647 (clone JK1B-1; biolegend), anti-IL-6-FITC (clone MQ2-13A5; BD), and anti-IL-1ra-PE (clone AS17; BD). Cytokine expression was detected on a cellular level by using flow cytometric analysis. CD68 was used for identification of monocytes after stimulation, which yielded better separation of monocytes than CD14 after stimulation.

### In vitro stimulation of whole blood with Toll-like receptor and nucleotide-binding oligomerization domain ligands and detection of interleukin-10 production by enzyme-linked immunosorbent assay

To determine IL-10 production, heparinised whole blood was mixed 1:2 with RPMI supplemented with 10 % fetal calf serum and stimulated without and with the respective TLR and NOD ligands for 18 h. Supernatants were harvested by centrifugation, snap-frozen, and stored at −20 °C until measurement of IL-10 by enzyme-linked immunosorbent assay (ELISA) (BD OptEIA, Heidelberg, Germany).

### Statistical analysis

Statistical analysis was performed by using GraphPad Prism 5 software (GraphPad Inc., La Jolla, CA, USA) and SPSS 19 software (IBM, Ehningen, Germany). Data are shown as individual measurements and medians. Non-parametric tests (Mann–Whitney *U* test and Kruskal-Wallis test and Dunn’s post test) were used for comparisons between groups. For variance analysis, we log-transformed data with uneven distribution and used linear regression analysis for metric variables and analysis of variance (ANOVA) for categorical variables.

## Results

### Expansion of classic monocytes in the peripheral blood of patients with axial spondyloarthritis

**Fig. 1:**
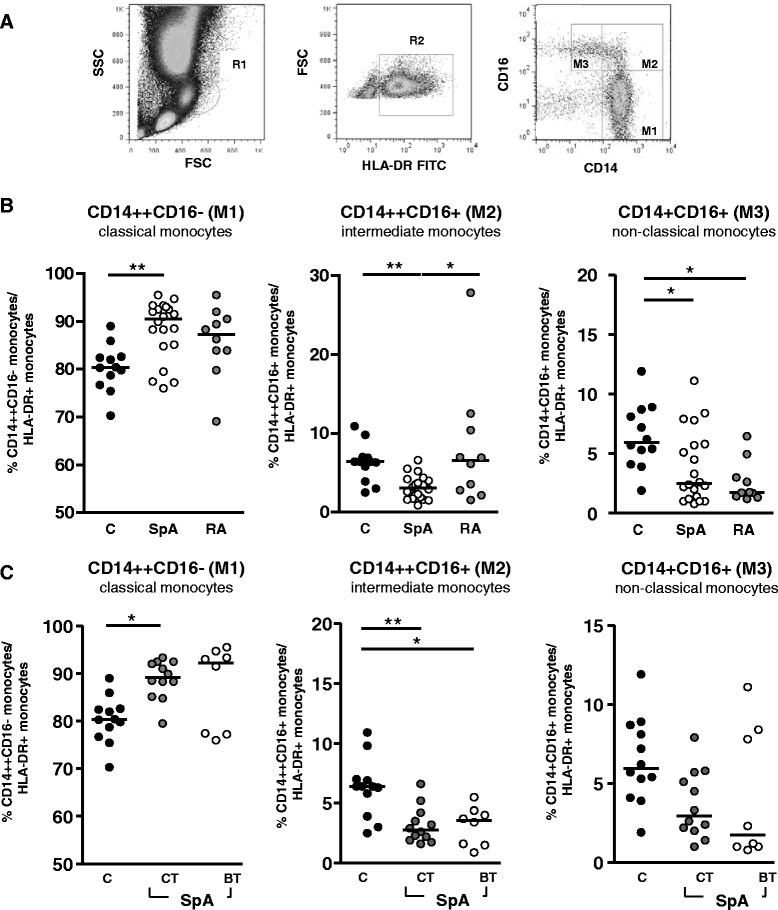
Elevated percentage of classic monocytes in patients with axial spondyloarthritis (axSpA). **a** The percentage of classic CD14^++^CD16^−^ (M1), intermediate CD14^++^CD16^+^ (M2), and non-classic CD14^+^CD16^+^ (M3) monocytes was determined by fluorescence-activated cell sorting in whole blood of controls, patients with axSpA, and patients with rheumatoid arthritis (RA). The percentage of classic, intermediate, and non-classic monocytes was determined among monocytes which were gated according to forward (FSC) and side (SSC) scatter (R1) and HLA-DR expression (R2). **b** The percentage of classic, intermediate, and non-classic monocytes (individual measurements and median) is shown from measurements in 12 controls (C), 20 patients with axSpA (SpA), and 12 patients with RA (RA). *P < 0.05, **P < 0.01 (Kruskal-Wallis test and Dunn’s post test). **c** The percentage of the respective populations in axSpA patients separated into patients under conventional treatment (SpA CT) (*n* = 12) and patients receiving biologics (i.e., tumor necrosis factor inhibitors) (SpA BT) (*n* = 8) compared with controls (c). *P < 0.05, **P < 0.01 (Kruskal-Wallis test and Dunn’s post test). *FITC* fluorescein isothiocyanate

To analyse the distribution of monocyte subsets (i.e., classic, intermediate, and non-classic monocytes) within the peripheral blood of axSpA patients in comparison with healthy controls and RA patients as an inflammatory control, monocytes were gated according to forward and side scatter and expression of HLA-DR (Fig. 1a). In patients with axSpA, the percentage of classic monocytes (CD14^++^CD16^−^) was significantly higher than in healthy controls (median of 90.5 % versus 80.4 %, *P* < 0.05; Fig. 1b) whereas intermediate (CD14^++^CD16^+^) and non-classic (CD14^+^CD16^+^) subsets were reduced in patients with axSpA (Fig. 1b). In patients with RA, non-classic monocytes were also reduced whereas differences in classic and intermediate monocyte subsets were not significantly different from controls. Subanalysis of axSpA patients grouped according to treatment into patients with conventional treatment (i.e., on demand or continuous NSAID treatment) or treatment with biologics showed significantly lower frequencies of intermediate CD14^++^CD16^+^ monocytes in patients under conventional as well as in patients treated with biologics compared with healthy controls, whereas the difference in frequencies of CD14^++^CD16^−^ monocytes reached statistical significance only in patients under standard therapy compared with healthy patients (*P* < 0.01) but not in patients under biological treatment (Fig. 1c).

The expression of HLA-DR as an antigen-presenting molecule, CD80 as a co-stimulatory molecule, CD163 as a scavenger receptor, CD64 as the Fc receptor, or CD115 as an activation marker on monocytes showed no difference between axSpA patients and controls (*P* > 0.05; data not shown).

### In vivo pre-activation of monocytes of axial spondyloarthritis patients according to proinflammatory cytokine production

**Fig. 2:**
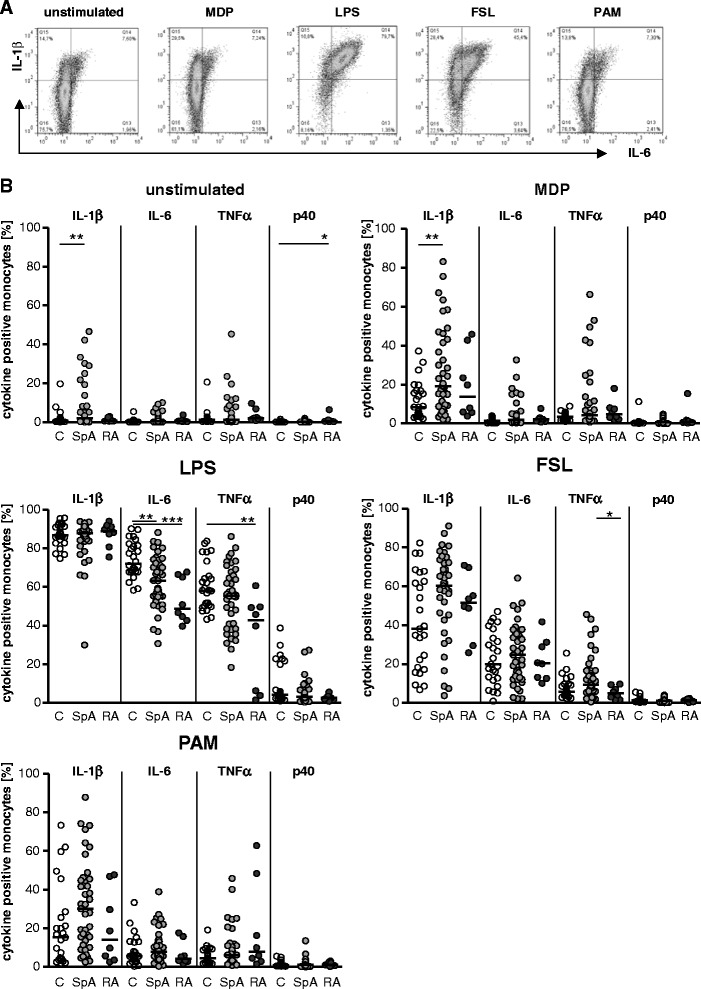
Elevated spontaneous and muramyl dipeptide (MDP)-induced proinflammatory cytokine production by monocytes from patients with axial spondyloarthritis (axSpA). Cytokine production by monocytes after stimulation of whole blood without (unstimulated) or with MDP, lipopolysaccharide (LPS), fibroblast-stimulating lipopeptid-1 (FSL), and PAM_3_CSK_4_ (PAM) for 5 h with brefeldin A added for the last 3 h. Cytokine production was analysed after intracellular staining by fluorescence-activated cell sorting. Monocytes were identified according to CD68 expression. **a** A representative example of cytokine production of CD68^+^ monocytes of a patient with axSpA in whole blood without stimulation and stimulation with the respective stimuli is shown. **b** The percentage of cytokine-positive cells among CD68^+^ monocytes (individual measurement and median) in response to the different stimuli in controls (C) (*n* = 26), patients with axSpA (SpA) (*n* = 42), and patients with rheumatoid arthritis (RA) (*n* = 8) is given. *P < 0.05; **P < 0.01 (Kruskal-Wallis test and Dunn’s post test). *IL* interleukin, *TNFα* tumour necrosis factor alpha

To determine spontaneous as well as TLR and NOD ligand-induced production of cytokines by monocytes, we incubated whole blood of 42 patients with axSpA (32 male, 10 female; mean age of 37.2 ± 9.8 years), 8 patients with RA (2 male, 6 female; mean age of 59.7 ± 18.8 years; all patients on prednisolone and DMARD treatment, no treatment with biologics), and 26 controls (13 male; 13 female; mean age of 35.4 ± 7.1 years) without any stimulator or added 2.5 μg/ml MDP (NOD-2 ligand), 100 ng/ml LPS (TLR4 ligand), 1 μg/ml FSL-1 (TLR2/6 ligand), or 5 μg/ml PAM_3_CSK_4_ (TLR1/2 ligand). Cytokine expression was determined in monocytes which were identified according to CD68 expression (Fig. 2a).

The percentage of monocytes spontaneously producing IL-1β was significantly higher in axSpA patients compared with healthy controls (Fig. 2b). A similar non-significant trend toward higher production was observed for IL-6 and TNFα production in patients with axSpA, whereas the IL-12/23p40 production was not increased.

Stimulation with MDP and also stimulation with FSL-1 elicited a higher cytokine production by monocytes, particularly of IL-1β, but also to lesser extent of IL-6 and TNFα, in patients with axSpA (Fig. 2b). For instance, after MDP stimulation, 19.2 % (median) of the monocytes of patients with axSpA versus 8 % of monocytes of healthy controls (*P* < 0.01) produced IL-1β. In contrast, IL-12/23 p40 production by monocytes was low (i.e., below 5 %) after MDP and FSL stimulation and was without difference between axSpA patients and healthy controls. In contrast, patients with RA did not show an elevated spontaneous or MDP- or FSL-induced production of proinflammatory cytokines, despite a minimal but significant increase in the percentage of IL-12/23p40-producting monocytes in unstimulated cultures. In contrast, no difference was observed between axSpA patients and controls in IL-1β and TNFα production in response to LPS, which elicited the highest cytokine response in monocytes. In patients with axSpA, more than 80 % of blood monocytes produced IL-1β in response to LPS and about 60 % produced TNFα, which was not different from controls. Also, on a per-cell basis, LPS elicited higher responses than MDP, FSL-1, or PAM_3_CSK_4_ as indicated by the higher mean fluorescence intensity of the IL-1β staining (Fig. 2a).

However, LPS stimulation resulted in significantly less IL-6-producing monocytes in axSpA patients compared with controls (median of 63.2 % versus 72.1 %; *P* < 0.01; Fig. 2b). Also, in patients with RA, LPS-induced IL-6 and even TNFα production was reduced in comparison with healthy controls (Fig. 2b). No significant difference between axSpA patients and controls was found in monocytic cytokine production in response to PAM_3_CSK_4_ stimulation (Fig. 2b).

The elevated spontaneous and MDP, and FSL-1, induced proinflammatory cytokine production by monocytes of patients with axSpA suggests in vivo pre-activation of monocytes in these patients.

### Elevated frequency of monocytes spontaneously producing interleukin-1 receptor antagonist in axial spondyloarthritis and rheumatoid arthritis patients

To determine whether the preactivation of monocytes in patients with axSpA is counterbalanced by an induction of anti-inflammatory mediators, we analysed the production of IL-1ra and IL-10. IL-1ra was detected by intracellular staining and FACS, whereas IL-10 which is induced at later time points after activation was measured by ELISA in the supernatant of whole blood stimulations performed in the absence of BFA. (Such cultures were performed only in axSpA patients and controls.)

**Fig. 3:**
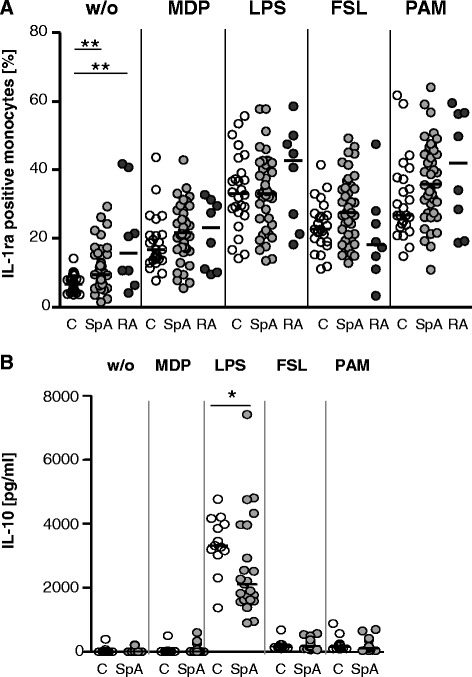
**a** Elevated spontaneous production of interleukin-1 receptor antagonist (IL-1ra) by monocytes of patients with axial spondyloarthritis (axSpA) and rheumatoid arthritis (RA). The percentage of IL-1ra-producing monocytes among CD68^+^ monocytes was determined as in Fig. 2. The percentage of cytokine-positive cells (individual measurement and median) in response to the different stimuli in controls (C) (*n* = 26), patients with axSpA (SpA) (*n* = 42), and patients with RA (RA) (*n* = 8) is given. **P < 0.01 (Kruskal-Wallis test and Dunn’s post test). Whole blood of patients with axSpA (SpA) (*n* = 26) and controls (C) (*n* = 13) was stimulated without or with indicated stimulators for 18 h. **b** IL-10 was measured in the supernatant by enzyme-linked immunosorbent assay. *P < 0.05 (Mann–Whitney *U* test). *FSL* fibroblast-stimulating lipopeptid-1, *LPS* lipopolysaccharide, *MDP* muramyl dipeptide, *PAM* PAM_3_CSK_4_, *w/o* without

The percentage of monocytes spontaneously producing IL-1ra was significantly elevated in axSpA patients but also RA patients compared with healthy controls (Fig. 3a). In contrast, no difference was found after stimulation with the different stimuli in the percentage of IL-1ra-producing monocytes between axSpA or RA patients and controls.

The IL-10 production induced by LPS was significantly lower in axSpA patients compared with controls (median of 1860 versus 3314 pg/ml; *P* < 0.05). In contrast, no difference was found in spontaneous or MDP-, FSL-1-, or PAM_3_CSK_4_-induced IL-10 production between axSpA patients and controls (Fig. 3).

### Analysis of association of monocytic cytokine production with clinical parameters and treatment in patients with axial spondyloarthritis

To determine the association between cytokine production and the state of disease progression according to the presence of radiographic sacroiliitis or the treatment, we log-transformed the data of patients with axSpA and performed a univariate variance analysis. This analysis (ANOVA) revealed association of spontaneous cytokine production (IL-1β, *P* = 0.009; IL-6, *P* = 0.03; TNFα, *P* = 0.012) and FSL-1-induced IL-6 production (*P* = 0.007) with therapy—conventional treatment versus treatment with biologics (i.e., TNFα inhibitors)—but no association with disease state (i.e., presence of radiographic sacroiliitis).

### Monocytic pre-activation is confined to axial spondyloarthritis patients under conventional treatment

**Fig. 4:**
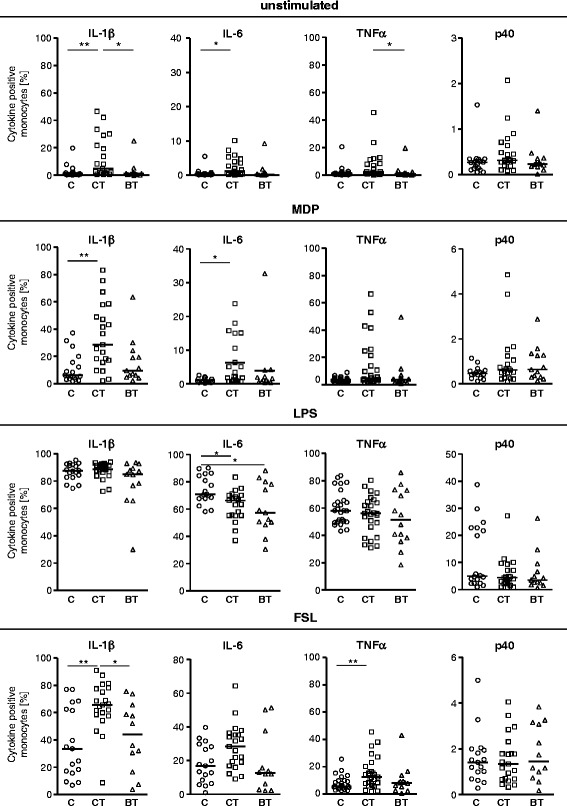
Disturbed cytokine responses are found predominately in axial spondyloarthritis patients under conventional treatment. Subanalysis of monocytic cytokine production (Fig. 3) in patients stratified according to treatment into a group of patients receiving conventional treatment spondyloarthritis (CT) (*n* = 20) and patients receiving biological treatment (i.e., TNF inhibitor treatment) (BT) (*n* = 12) compared with healthy controls (C) (*n* = 17). *P < 0.05; **P < 0.01 (Kruskal-Wallis test and Dunn’s post test). *FSL* fibroblast-stimulating lipopeptid-1, *IL* interleukin, *LPS* lipopolysaccharide, *MDP* muramyl dipeptide, *TNFα* tumour necrosis factor alpha

As the previous analysis suggested an impact of the therapy on the cytokine responses by monocytes, we performed a subanalysis of the data by stratifying the axSpA patients according to therapy into patients receiving conventional treatment and patients receiving TNFα inhibitors. This subanalysis revealed that elevated spontaneous production of IL-1β, IL-6, and TNFα was confined to axSpA patients under conventional treatment (Fig. 4). In this group, the percentage of monocytes spontaneously producing IL-1β and TNFα was significantly higher than in patients under TNFα inhibitor (i.e., biological treatment) (*P* < 0.05).

Also, the enhanced production of proinflammatory cytokines in response to MDP and FSL-1 was restricted to the group of patients under conventional treatment (Fig. 4). In this group of patients, the percentage of monocytes producing IL-1β and IL-6 after MDP and FSL-1 stimulation was significantly higher than in controls even after subtraction of the percentage of monocytes spontaneously producing these cytokines (data not shown). In contrast, no difference was found between patients under conventional treatment and patients under TNFα inhibitor treatment in the percentage of monocytes producing IL-12/23p40 (spontaneous or MDP- or FSL-induced).

Interestingly, LPS-induced cytokine responses did not differ between both groups of patients; that is, IL-6 (Fig. 4) and IL-10 production (data not shown) was reduced in both groups of patients compared with controls (*P* < 0.05). Moreover, there was no difference in the percentage of IL-1ra-producing monocytes between patients under conventional treatment and patients under TNFα inhibitor treatment (data not shown).

To determine the association between monocytic cytokine production and clinical parameters such as inflammatory parameters, presence of radiographic sacroiliitis, and disease activity in patients with axSpA under conventional treatment, we performed an ANOVA and linear regression analysis. In this analysis, monocytic cytokine production (unstimulated and MDP-, LPS-, and FSL-1-stimulated) was not associated with CRP, but a strong association of monocytic cytokine production was observed with BASDAI (Table 1).

**Table 1:** *P* values from univariate analysis of variance between clinical variables and cytokine responses in axial spondyloarthritis patients (*n* = 20) under standard treatment (*P* values, log-transformed data)

	Sacroiliitis	CRP	BASDAI
Unstimulated			
IL-1	0.980	0.539	0.011
IL-6	0.604	0.829	0.025
TNF	0.856	0.984	0.006
IL-1RA	0.299	0.449	0.181
IL-10	0.488	0.584	0.318
MDP			
IL-1	0.300	0.126	0.040
IL-6	0.693	0.698	0.034
TNF	0.439	0.963	0.007
IL-1RA	0.098	0.433	0.010
IL-10	0.634	0.812	0.177
LPS			
IL-1	0.888	0.584	0.005
IL-6	0.546	0.625	0.029
TNF	0.692	0.516	0.001
IL-1RA	0.543	0.744	0.048
IL-10	0.691	0.742	0.010
FSL			
IL-1	0.492	0.470	0.642
IL-6	0.389	0.483	0.669
TNF	0.861	0.918	0.669
IL-1RA	0.227	0.390	0.040
IL-10	0.714	0.199	0.037

*CRP* C-reactive protein, *BASDAI* Bath ankylosing spondylitis disease activity index, *IL* interleukin, *TNF* tumor necrosis factor, *MDP* muramyl dipeptide, *LPS* lipopolysaccharide, *FSL* fibroblast-stimulating lipopeptid

### Difference in cytokine production by monocytes is not due to differences in Toll-like receptor expression in patients with axial spondyloarthritis

**Fig. 5:**
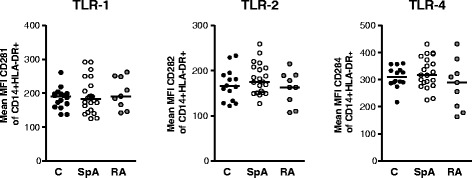
No difference in Toll-like receptor (TLR) 1, 2, and 4 expression on CD14^+^ monocytes between axial spondyloarthritis patients and controls. The expression of TLR-1, TLR-2, and TLR-4 on monocytes was determined in healthy controls (C) (*n* = 16), patients with axial spondyloarthritis (SpA) (*n* = 22), and patients with rheumatoid arthritis (RA) (*n* = 9) by surface staining and fluorescence-activated cell sorting and is given as the mean fluorescence intensity (MFI). Monocytes were identified according to CD14 expression

To determine whether differences in TLR ligand-induced monocytic cytokine production between axSpA patients and controls are due to differences in expression of TLR ligands, we analysed the expression of TLR 1, 2, and 4 on monocytes. No difference was found in surface expression of either of the receptors on monocytes in axSpA patients and RA patients compared with controls (Fig. 5).

## Discussion

In this study, we observed an elevated frequency of classic (i.e., CD14^++^CD16^−^) monocytes and a reduction in non-classic (i.e., CD14^+^CD16^+^) monocytes in peripheral blood of patients with axSpA and this is in accordance with a study by Surdacki et al. [11]. TNFα inhibitor treatment did not reverse these phenotypic differences. In patients with RA, we also observed a trend toward an increase of classic monocytes at the expense of non-classic monocytes. This is in line with data from Klimek et al. [12] but in contrast to other studies which reported an expansion of CD14/CD16 double-positive (i.e., non-classic) monocytes in RA [13, 14]. These discrepancies are most likely related to differences in therapy; in particular, glucocorticoid therapy has been described to deplete CD14^+^CD16^+^ monocytes [15]. In patients with Crohn’s disease, an expansion of non-classic monocytes was described [16]. It was suggested that the imbalance between monocyte subsets in patients with Crohn’s disease is due to recruitment of the classic monocytes, which are more migratory than the non-classic monocytes, to sites of inflammation [16]. Therefore, the difference between axSpA and Crohn’s disease may be caused by differences in the extent of local inflammation which controls recruitment of monocytes. On the other hand, HLA-B27 could also affect the differentiation of monocyte subsets and promote the differentiation of classic monocytes.

Thiesen et al. analysed cytokine production of sorted human monocyte subsets and found that classic monocytes, though considered more phagocytic and less inflammatory, produced higher levels of proinflammatory cytokines than the non-classic monocytes [16]. Interestingly, we observed enhanced spontaneous as well as MDP- and FSL-1-induced production of proinflammatory cytokines in patients with axSpA and, according to the data by Thiesen et al., this could be related to the expansion of classic monocytes.

The enhanced cytokine production by monocytes of patients with axSpA in particular to suboptimal stimuli such as MDP and FSL-1, which require co-activating signals such as ineffective doses of LPS [17] or low levels of cytokines, suggests that monocytes of patients with axSpA are pre-activated in vivo. This is also suggested by mRNA expression profiles showing enhanced IL-1 receptor expression in blood cells of patients with axSpA [18] and by results of protein profiling of monocytes of axSpA patients that showed enhanced expression of proteins involved in TLR signaling, leukocyte extravasation, and other inflammatory pathways [8]. Genome association studies [19] and experimental [20] and clinical [21] data suggest an involvement of the IL-23-Th17 pathway in axSpA. However, the production of IL-12/23p40 production was rather low in peripheral blood monocytes in our study and with no difference between axSpA patients and controls. Even after strong stimulation with LPS, only about 5 % of the monocytes produced IL-12/23p40, suggesting that monocytes are not a major source of this cytokine. In fact, our own analysis of the bone marrow of facet joints of patients with AS revealed myeloperoxidase-positive cells and, to a lesser extent, macrophages and dendritic cells as sources of IL-23 [22].

In contrast to the enhanced cytokine responses to MDP and FSL-1, cytokine production in response to LPS was, at least partially, reduced in patients with axSpA. Reduced LPS responses are observed in endotoxin tolerance which is induced by pre-exposure of monocytes to low doses of LPS. However, the suppression of proinflammatory cytokine responses by monocytes of patients with axSpA is rather minor and accompanied by a strong suppression of anti-inflammatory IL-10 production, whereas the state of endotoxin tolerance and monocyte paralysis, as for instance observed in patients with sepsis, is characterized by a strongly suppressed proinflammatory response and partly preserved IL-10 production [23]. Thus, the decent reduction of cytokine responses to LPS which is the strongest of the tested stimuli may indicate exhaustion of monocytes after pre-activation rather than tolerization.

The enhanced spontaneous production of cytokines was restricted to axSpA patients under conventional treatment and is not found in axSpA patients under TNFα inhibitor treatment or in RA patients. However, LPS-induced IL-6 and IL-10 production was also reduced in axSpA patients who received TNFα inhibitor. This could be attributable to the presence of TNFα inhibitors in the whole blood stimulation which may neutralize some autocrine effects of TNFα produced during early activation. In particular, IL-10 depends on autocrine production of TNFα [7]. Also, in patients with RA, LPS-induced production of IL-6 and TNFα was reduced and this may also be related to glucocorticoid therapy [24].

Differences in cytokine responses to TLR ligands may be caused by differential expression of TLRs. In fact, enhanced expression of TLR 4 on blood monocytes has been described in patients with SpA [25]. However, in our study, no differences were found in the expression of TLR 1, 2, and 4 in axSpA patients compared with controls and this might be due to technical differences as we analysed expression in whole blood as compared with isolated peripheral blood mononuclear cells and used a different gating strategy.

Interestingly, stimulation of bone marrow cells and splenocytes from HLA-B27/huβ2m transgenic rats with TNFα [26] or zymosan or *Mycobacterium tuberculosis* also resulted in elevated production of IL-1α and IL-1β compared with control rats, indicating that the enhanced responsiveness of axSpA patients to bacterial triggers may be related to HLA-B27. On the other hand, the expansion of classic monocytes and in vivo pre-activation of monocytes in patients with axSpA could also be the result of in vivo activation by cytokines released by local inflammation or by bacterial components such as LPS which may translocate during intestinal inflammation as mucosal inflammation is a common feature in axSpA. Thus, about 5 % of patients with AS also suffer from overt inflammatory bowel disease [27] and about 50 % show microscopic gut lesions in the absence of gastrointestinal discomfort [28].

Delineation of potential triggers of the spontaneous cytokine production is difficult since cellular responses of monocytes to LPS and cytokines such as TNFα are strongly overlapping [29]. Only some genes such as apoptosis-related genes are selectively induced in monocytes by LPS in vitro [29] and analysis of their expression might provide clues on the activating signal of monocytes in patients with axSpA.

TNFα inhibitor treatment of axSpA normalized the functional state of monocytes as elevated cytokine responses were confined to axSpA patients under conventional treatment. The fact that MDP- and FSL-1-induced cytokine production was unaffected under TNFα inhibitor treatment suggests that the effect of TNFα inhibitor treatment is an in vivo effect rather than an in vitro effect. Thus, the TNFα inhibitor treatment may interfere with the triggers of monocytic cytokine production such as local cytokine production or bacterial antigen translocation.

Interestingly, we found that spontaneous MDP- and LPS-induced production of cytokines correlated with disease activity. As CRP correlated neither with BASDAI nor with the monocytic cytokine production, monocyte activation might be a more sensitive indicator of inflammation than CRP. However, the monocytic pre-activation, particularly the spontaneous production of IL-1β as well as IL-6, does not seem to be directly pathogenic as blockade of IL-1 [30] as well as IL-6 [31] is of limited or no efficacy in reducing disease activity in AS. The enhanced IL-1β and IL-6 production by monocytes rather seems to be an indicator of systemic innate immune activation while other immune functions of monocytes or other cell types may exert the pathogenic effects.

## Conclusions

This study reveals in vivo pre-activation of monocytes in axSpA patients indicated by enhanced spontaneous as well as MDP- and FSL-induced cytokine production by monocytes which correlates with BASDAI. This in vivo pre-activation is confined to patients under conventional therapy and reverted under TNFα inhibitor treatment. Further work should delineate the triggers of this systemic activation of innate cells.

## Abbreviations

ANOVA: Analysis of variance
AS: Ankylosing spondylitis
axSpA: Axial spondyloarthritis
BASDAI: Bath ankylosing spondylitis disease activity index
BFA: Brefeldin A
CRP: C-reactive protein
DMARD: Disease-modifying anti-rheumatic drug
ELISA: Enzyme-linked immunosorbent assay
FACS: Fluorescence-activated cell sorting
FITC: Fluorescein isothiocyanate
FSL-1: Fibroblast-stimulating lipopeptid-1
IL: Interleukin
LPS: Lipopolysacchride
MDP: Muramyl dipeptide
NOD: Nucleotide oligomerization domain
NSAID: Non-steroidal anti-inflammatory drug
RA: Rheumatoid arthritis
TLR: Toll-like-receptor
TNFα: Tumour necrosis factor alpha
